# Supplementary material for: Perceived paternal emotional fertility intention and its correlates in Ethiopia among a cohort of pregnant women: Community based longitudinal survey; A secondary data analysis of the 2019/20 baseline survey
Source: PLoS One. 2025 Feb 27;20(2):e0318654. doi: 10.1371/journal.pone.0318654 (PMC11867388; doi:10.1371/journal.pone.0318654)
Supplement: Attachment — Submitted filename: Paternal 21_ Point by Point Response_19 Sep 2024.pdf [file pone.0318654.s001.pdf]

Date: 19 Sep 2024

To Plos One

Subject: Point by Point Response

Manuscript Id: PONE-D-24-25872
Title Perceived Paternal Fertility Emotion and Its correlates in Ethiopia among a Cohort of Pregnant Women: Community based Longitudinal Survey
Journal: PLOS ONE

The authors very much appreciate for the constructive and valuable comments provided by the reviewers which guided the authors to improve the quality of the revised manuscript. We appreciated all comments and used as an input to improve the quality of the manuscript without such feedback the revised manuscript would not take its current form. We also extend our sincere acknowledgment to handling the editor for giving us the opportunity to further improve the quality of the manuscript so that it will suitable for publication.

Response to Reviewer one valuable Concerns:

Reviewers' comments:

Response to Reviewer 1:

Reviewer #1:

General Response: We very much appreciate the reviewer's constructive feedback and suggestion. We have attempted to provide responses for the specific queries.

Comment 1: Title: - Please include that this is a secondary data analysis and specify the year of the study.

Response 1: We appreciate the comment and comment addressed in the revised version.
Revised Title Perceived Paternal Fertility Emotion and Its correlates in Ethiopia among a Cohort of Pregnant Women: Community based Longitudinal Survey; **A secondary data analysis of 2019/20 Baseline Survey**

Abstract:

Comment 2: - Results Section: There is an inconsistency regarding higher birth order. The authors report that having 3 to 12 children (AOR=0.14) is associated with higher birth order, yet at the end of the results section, they state that higher birth order was a significant predictor of being happy about the index pregnancy. Please clarify this inconsistency.

Response 2: We have double checked the twos sections and found no discrepancy for the very happy categories of the outcome since we have two categories for the outcome to be compared internally with the base category. In both sections higher birth order lower perceived paternal fertility emotion between the what is stated in the result part of the abstract and the main result part: page 11 paragraph one: summary and paragraph four narration about the specific variable. This applies for the very happy category.

Note that; we have two upper level categories; the very happy and a sort of happy category for ARRR is reported as compared with the base category of the outcome. 3 to 12 children (AOR=0.14): this is for the very happy category while the later stated as significant predictors and the effect measure for the sort of happy category to which the specific ARRR is plugged in with the abstract in the revised version.

Background:

Comment 3: - The introductory paragraph could benefit from restructuring. I recommend following a funnel approach to better organize the background information.

Response 3: Comment accepted and efforts were made to rearrange the background information so that it will at least resembles the funnel approach suggested by the reviewer.

Language and Style:

Comment 4: - The manuscript would benefit from improvements in English language grammar and overall writing style.

Response 4: We have attempted to get supported from English Language Professional Colleague.

Methodology:

Comment 5: - Sample Population: Provide additional details about the PMA (Performance Monitoring for Action) including the sampling process and the type of data collected.

Response 5: PMA employed a two cluster stage sampling; in the first stage enumeration areas (EA) were selected. In each selected EA fresh listing and census was conducted to screen and enroll pregnant women

and less than 6 weeks postpartum women by the time of the baseline survey those this study was restricted to only 2,115 pregnant women from 217 enumerations areas who were married and/or living together as a partner by the time of the base line survey and who completed the baseline female questionnaire. Following enrollment the Female baseline questionnaire was administered. In the female baseline questionnaire women were asked about their antenatal care sought thus far in their idex pregnancy, partner support and perceived community encouragement on the use the three main domains of the maternal and newborn care components, their reproductive and sexual history, their birth preparedness and complication readiness, about their agreement and disagreement on girls and women empowerment towards contraceptive use; women sexual and reproductive issues, contraceptive use history and there fertility intention. Women were asked how they themselves did felt when learned the index pregnancy. Most importantly to this study, they were also asked how their husband felt when their husbands and/or partners learned their index pregnancy, which regarded as women perceived paternal fertility emotion towards the index pregnancy which is the main outcome variable in this study.

Comment 6: - Study Design: The authors describe this study as a cohort study. However, it is unclear if the pregnant women were followed over time to assess any changes in their partner's emotions towards the pregnancy. Please clarify this aspect.

Response 6: We appreciate the comment. Sure the actual study continued to one postpartum with 6 weeks, 6 months and one year postpartum follow up after the baseline survey. This study mainly rely on the question how their husband felt when leaned the index pregnancy, neither the husbands were asked. This why we use the term perceived. Hence, we include the following limitations:

Though reliance on self-reported data by asking the women to learn about how their husband felt about the index pregnancy to measure on paternal emotional fertility intention which lead to potential biases particularity; such findings offer meaningful insights that advance the field of study. However, further research is needed to validate and build upon these initial results.

For your information women were asked what did the feel if they became pregnant in the follow up interviews.

Comment 7: - Data Collection: The rationale for accessing data at 6 weeks postpartum is unclear. Were participants questioned about their pregnancies post-delivery? The absence of reporting on this second data point suggests that this might be more accurately described as a cross-sectional study, where a group of women was surveyed about their pregnancies at a single point in time.

Response 7: Thank you very much for the question posed, accessing six weeks postpartum date set was mention in the Ethical consideration section, page 8. For your information, this data set were not used in this particular analysis and instead only the baseline cross-sectional data set from the prospective study were analyzed!

Sample Size:
Comment 8: - Include a flowchart diagram illustrating the sample selection process and how the final sample size was determined.

Response 8: Two thousand two hundred thirty nine **(**2,239) women provided response for the When your partner found out you were pregnant, how did he feel, 124 were exclude from the analysis due to 3 women provided no response, 46 reported do not know, 57 reported that they have not told to their partner and 18 of them reported they have no partner. Hence, the final sample size was 2115.

I**ndependent Variables:**

Comment 9: - Please enumerate the individual-level and enumeration area-level variables used in this study.

Response 9: Comment accepted and the following revision was made in the revised manuscript version.

Sociodemographic: age of women, educational status, wealth index, marital status and religion of the women. RH characteristics: birth order, marriage type, marriage history, fertility intention, health service use related such as contraceptive ever use history and planned birth attendant and planed place of delivery for the index child. The integral enumerating area level variables were region and place of residence. Note that derived enumeration area level variables were EA level wealth and proportion of women with secondary education and above. Here below is the list of independent variables included in this study along with the final recode categories,

**Variable name and Categories**	
Age category	15-19 years<br>20-24 years<br>25-29 years<br>30-34 years<br>35-39 years<br>40-49 years
Educational Status	No Formal Education<br>Primary Education<br>Secondary Plus
Religion	Other<br>Orthodox<br>Protestant<br>Muslim
Wealth Index	Lowest quintile<br>Lower quintile<br>Middle quintile<br>Higher quintile<br>Highest quintile

Variable name and Categories	
Parity	No Child<br>1_2 Children<br>3_12 Children
Marriage History	Only_ Once<br>More than_ Once
Marriage Type	Monogamy<br>Polygamy
Fertility Intension	Undecided/DKN<br>Have a/another child<br>No more or prefer no child
Marital Status	Married<br>Living With A partner<br>Widowed/Separated
Contraceptive Ever Use	No<br>Yes
Residence	Urban<br>Rural
Region	Tigray and Afar<br>Amhara<br>Oromiya<br>SNNPR<br>Addis Ababa
Desired Birth Attendant	No One/ND<br>HP<br>Family Member
Desired Delivery Place	Home/ND<br>Government Private HF

Discussion:

Comment 10: - The second paragraph is unnecessary and poorly written.

Response 10: Comment accepted and efforts were made to amend the second paragraph.

Comment 11: - Overall, the discussion section is underdeveloped and requires significant improvement.

Response 11: Comment accepted and efforts were made to enrich the discussion part, which is all related with the dearth of literature directly fit the study measurement approach. Efforts were made to enrich the discussion part in the revised version. Additional empirical evidence was sought and cited.

Response to Reviewer 2:

Reviewer #2: Dear authors,

Congratulations on your study. The subject is important and relevant to outlining care for pregnant women and their partners. Some suggestions listed below could strengthen your manuscript.

General Response: We appreciate the encouragement and for the reviewer´s kind words. We have attempted to provide responses for the specific queries.

In the introduction:

Comment 1: 1. I was unsure if 'Intention to conceive' is the same as 'Intention to fertility'? You talk about these expressions and they seem to be synonyms, but they can be totally different when you investigate the meaning of the words. If you're referring to different processes, I'd gently suggest making that clearer in the text. A brief adjustment to the paragraph is sufficient.

Response 1: We considered the reviewers comment and preferred the term fertility intention over intention to conceive which is corrected in the revised version.

On the method:

Comment 2: 2. I'm understanding that the aim of the study was to determine the level of perceived paternal fertility emotion about the index pregnancy among a cohort of pregnant women and to identify their correlates for such variation, right? But when I read the method, I realize that the data was collected from women. I wonder if I've understood the objective correctly, because how can we measure the perception of fathers through mothers? Shouldn't this information be collected from men?

Response 2: We acknowledge this concern as it's related with outcome variable measurement. As you rightly mentioned, It could have been better had the information was collected from men. Since Such data were scarce and we relied on secondary data we used the term perceived paternal fertility intention as his feeling towards the index pregnancy were measured by asking his wife which was used as a proxy measure. This is acknowledge as a limitation of this study.

This study is not spared of limitations. To start with though reliance on self-reported data by asking the women to learn about how their husband felt about the index pregnancy to measure on paternal emotional fertility intention which lead to potential biases; such findings offer meaningful insights which is important and relevant to outlining care for pregnant women and their partners. However, further research is needed to

validate and build upon these initial results. In addition, because of PMA 2020 didn't collect information on variables such as husband desired number of children, husband employment and women employment were not measured in this study.

Comment 3: 3. What was the design of the study presented in this article? Was it a cross-sectional study with qualitative data? or quantitative? or mixed method? I strongly suggest signaling this right at the beginning of the method passage.

Response 3: Comment accepted and the following introduction signaling the specific data source and design used in this study plugged in: This study used the baseline cross-sectional data from prospective cohort study with 6 weeks, 6 months and one postpartum follow up interviews apart from the baseline cross sectional data used for this study. The data was collected from pregnant women in Ethiopia from six regions: namely: Addis Ababa, Afar, Amhara, Oromia, SNNPR and Tigray.

Comment 4: 4. Although the data has already been published, providing a brief contextualization of the study makes it easier to understand. I'm not saying that you have to write everything down in exhaustive detail. Just contextualize it simply and objectively: this is a cross-sectional study of the baseline of a prospective cohort. The data was collected from pregnant women in X health centres in Ethiopia....

Response 4: We appreciate the comment and the following introduction at the beginning of the methods part was used: this study used the community based baseline cross-sectional data from prospective cohort study with 6 weeks, 6 months and one postpartum follow ups interviews. The data was collected from pregnant women in Ethiopia from six regions: namely: Addis Ababa, Afar, Amhara, Oromia, SNNPR and Tigray.

Comment 5: 5. Where you reference the details of the Ethiopia PMA, I suggest that you leave only the citation of the study and move the reference to the references section. For example: 'A detailed description of the Ethiopia PMA protocol has been published previously (citation)' or 'Information regarding the selection of the sample and the units is described in detail in the Ethiopia PMA protocol (citation)'.

Response 5: Comment accepted and corrected in the revised version of the manuscript.

Comment 6: 6. I kindly suggest that you objectively describe which sample was used in this article. It doesn't matter if you recruited people with a different profile at other points in the study. What matters is that you make it clear what the inclusion and sampling criteria were for the section of the study you are presenting here. For example: puerperal women were recruited throughout the study, but only pregnant women were recruited at baseline. So you can go straight to the information that the sample consists of pregnant women.

Response 6: we acknowledge your concerns and address this very important concern by including expiation in the methods part within the revised version The study started by recruiting and enrolling pregnant women and puerperal women less than six weeks which was followed admistrising the female baseline questionnaire.

Then these panel of women were interviewed at 6 weeks, 6 months and one year postpartum as follow up interviews. However, this study further analyzed and present data from the baseline survey

Results:
The findings are very good. The quality of the data is excellent, given the size of the sample and the categories of responses. It's positively surprising!

Comment 7: 7. It would be very comfortable for the reader if you could include the number of participants who were included in the analysis in the second line of the results. Even if it is already well described in the method, just for comfort in reading and interpreting. '2115 women took part in the study. The proportion of parental emotion....' is enough.

Response 7: Comment accepted and revision made in the revised version. Responses on how pregnant women perceived paternal fertility intention (what their husbands and/or partners did they felt when learned the index pregnancy) by the time they were pregnant from a total of 2,115 pregnant women were further analyzed in this study.

Comment 8: 8. In the passage: Correlates of Perceived Paternal Fertility Emotion about the Index Pregnancy among a panel of pregnant women and its correlates in Ethiopia, Evidence from Cohort One Baseline Data, 2019 to 20. The first sentence "This study investigated factors affecting perceived paternal fertility emotion about the index pregnancy among a cohort of pregnant when their husband and/or partner learned their wife´s index pregnancy" fits better at the end of the introduction or in an objective passage. You don't have to rewrite it in the results because it should be clear in the method. So I truly suggest you exclude this sentence in this paragraph, but consider bringing it to the end of the introduction or at the beginning of the method.

Response 8: Thank you very much for your concern, we have deleted in here since we have felt that conclude the introduction part in a similar manner. Since our intention of using this narration is addressed by your comment above, comment 7, we are comfortable to delete this piece of sentence.

Discussion:
Comment 9: 9. In the second paragraph of the discussion you cited "the authors" two times. It is not clear if you are talking about the authors of the dataset or if you are talking about you (authors of the manuscript) in a third voice. If you are talking about the dataset's authors, I strongly suggest you provide the citation in this paragraph. If you are not, I suggest making it clear that you are discussing your findings. This passage is a little confusing.

Response 9: Its refers the authors of this particular paper and edition was made in the revised version.

Comment 10: 10. What limitations did the study have? Think about reiterating that paternal feelings were assessed through women's reports and are therefore subject to bias. How can someone else tell what I'm feeling?
Conclusion:

Response 10: This study is not spared of limitations. To start with though reliance on self-reported data by asking the women to learn about how their husband felt about the index pregnancy to measure on paternal emotional fertility intention which lead to potential biases; such findings offer meaningful insights which is important and relevant to outlining care for pregnant women and their partners. However, further research is needed to validate and build upon these initial results. Moreover, because of PMA 2020 didn't collect information on variables such as husband desired number of children, husband employment and women employment were not measured in this study.

Comment 11: 11. I totally agree with your point of view, but you should respond to your study's objective here. The other statements about what to do with these findings must be discussed in the discussion passage.

Response 11: We very much appreciate and we have attempted to address our objectives and looked its implication in some instances.

Comment 12: 12. The English is a little hard to follow. An English language review would be nice.

Response 12: We have attempted to get support from English Language Professional Colleague.

Additionally;

- The ethical consideration from the declaration section was removed and the one in the methods part was kept.
- Figures and tables from the body of the result were deleted with proper citation in the textual narration and uploaded as supporting information file.
- As the same time description on the list of tables and figures was provided after the reference.
- Caption was added for figure one below the figure though figure one has a capitation embedded within the figure.
