# Supplementary material for: Perceived paternal emotional fertility intention and its correlates in Ethiopia among a cohort of pregnant women: Community based longitudinal survey; A secondary data analysis of the 2019/20 baseline survey
Source: PLoS One. 2025 Feb 27;20(2):e0318654. doi: 10.1371/journal.pone.0318654 (PMC11867388; doi:10.1371/journal.pone.0318654)
Supplement: Attachment — Submitted filename: R3_Paternal 21_ Point by Point_Dec 2024.docx [file pone.0318654.s002.docx]

Date: 04 Dec 2024

To Plos One

Subject: Point by Point Response

Manuscript Id: PONE-D-24-25872

# Title **Perceived Paternal Emotional Fertility Intention and Its correlates in Ethiopia among a Cohort of Pregnant Women: Community based Longitudinal Survey; A Secondary Data Analysis of 2019/20 Baseline Survey**

Journal: PLOS ONE

The authors very much appreciate for the constructive and valuable comments provided by the reviewers which guided the authors to improve the quality of the revised manuscript. We appreciated all comments and used as an input to improve the quality of the manuscript without such feedback the revised manuscript would not take its current form. We also extend our sincere acknowledgment to handling the editor for giving us the opportunity to further improve the quality of the manuscript so that it will suitable for publication.

Response to Reviewer one valuable Concerns:

Reviewers' comments:

Please use the space provided to explain your answers to the questions above. You may also include additional comments for the author, including concerns about dual publication, research ethics, or publication ethics. (Please upload your review as an attachment if it exceeds 20,000 characters)

Reviewer #1: Abstract

**Comment 1:** 1. The term "fertility emotion" should be defined briefly to ensure clarity for readers unfamiliar with the concept.

**Response 1:** The authors appreciate the comment and amendment made in the revised version. Revisions made accordingly.

**Comment 2:** 2. Clarify how the findings contribute to existing knowledge or practice.

**Response 2:** The authors appreciate the comment and amendment made in the revised version in the implication of a study. The existing justification has the significance of the study narrated within it.

Introduction

**Comment 3:** 1. Clearly articulate the specific research gap this study addresses. While it is implied that limited research exists in this area, explicit evidence (e.g., citing prior studies or lack thereof) would strengthen the justification.

**Response 3:** The authors appreciate the comment and amendment made in the revised version. As narrated in the background justification and picked up by the reviewer the later the explanation worked.

**Comment 4:** 2. The research objectives should be stated more explicitly. For example:
"This study aims to explore the perceived fertility emotions of male partners and examine their correlates among pregnant women in Ethiopia."

**Response 4:** The authors appreciate the comment and amendment made in the revised version the stated justification were made.

**Comment 5:** 3. Clearly define how "Paternal Fertility Emotion" concept was operationalized. Were validated tools used, or was a new tool developed? If a new tool was developed, describe the validation process.

**Response 5:** The authors appreciate the comment and amendment made in the revised version. The authors would appreciated this study can service a preliminary finding in the area which could be capitalized by further studies. The following recommendation was added to address the aforementioned concern: Further research aimed at exploring perceived paternal fertility emotions across different cultural or socio-economic groups. Additional studies are needed which come up with a direct measurement of paternal emotional fertility intention including validating this direct measurement tool to be developed.

Results
**Comment 6:** 1. Ensure consistency in terminology. For instance, if terms like "positive fertility emotion" or "negative fertility emotion" are used, define them clearly and use them consistently throughout the manuscript.

**Response 6:** In the result section particularly the factors related section to mention show the direction of the statistical association, meaning those factors the increased the odds of perceived paternal emotional fertility intensions and those factors which were found to lower the it.
Discussion

**Comment 7:** 1. Clearly explain how this study addresses the identified gap in knowledge.

**Response 7:** We appreciate the comment and the significance of the study is presented in the background part. Additional relevance has been added as implication of the study.

**Comment 8:** 2. Discuss the implications of these findings for maternal and paternal health policies or interventions in Ethiopia not a copy-paste of an implication from another study!! “The implication of this study is increasing men fertility knowledge and their emotional readiness as supported by a finding from a study Men with university education had better fertility knowledge than men without university education along with lifestyle adjustment before pregnancy to improve health and fertility (32)”

**Response 8:** The authors appreciate the comment and amendment made in the revised version.

The following additional argument has been added….In addition, More educated husbands tend to understand their wives communicate smoothly and help them while seeking person centered care during pregnancy and childbirth care (52). The other implication of the study finding was that it provided its share for developing and implementing males’ involvement strategy in during pregnancy, childbirth and related reproductive health service use.

**Comment 9:** 3. The limitations section should be expanded to address potential biases (e.g., social desirability bias in responses) and generalizability concerns.

**Response 9:** The authors appreciate the comment and amendment made in the revised version. The following has been expanded in the limitation part as per the comment from the reviewer.

… Potential biases such as social desirability bias as talking negative about once husband and/or partner has been a customer practice in our context. ….

.However, further research is needed to validate and build upon these initial results including studies which come with alternative and direct measures of paternal fertility emotional intention. In addition, because of PMA 2019/20 baseline survey didn’t collect information on variables such as husband desired number of children, husband employment and women employment were not measured in this study. Further research aimed at exploring perceived paternal fertility emotions across different cultural or socio-economic groups.
**Comment 10:** 4. Suggest specific areas for future research, such as exploring fertility emotions across different cultural or socio-economic groups.

**Response 10:** The authors appreciate the comment and amendment made in the revised version. See the

The following recommendation has been added.
Further research aimed at exploring perceived paternal fertility emotions across different cultural or socio-economic groups. Additional studies are needed which come up with a direct measurement of paternal emotional fertility intention.
